# Building a new ecosystem for digital-intelligent teaching of veterinary infectious diseases in southwest Chinese universities: theoretical framework and practical pathways

**DOI:** 10.3389/fvets.2026.1746110

**Published:** 2026-02-05

**Authors:** Xiao Wang, Meiquan Li, Yanli Du, Heng Yang, Jinshan Ran, Hongyan Zhang

**Affiliations:** College of Agriculture and Life Sciences, Kunming University, Kunming, China

**Keywords:** digital-intelligent teaching, practical pathways, universities in Southwest China, teaching model innovation, theoretical framework, veterinary infectious diseases

## Abstract

Traditional veterinary infectious disease education faces inherent limitations, particularly in presenting abstract mechanisms, complex epidemiology, and high-risk scenarios. To address these challenges and meet the World Organisation for Animal Health (OIE)’s capacity building requirements, digital-intelligent teaching is driving a paradigm shift from “knowledge transmission” to “competency development.” This study systematically constructs a clearly layered (macro-meso-micro) conceptual model for digital-intelligent teaching, hierarchically integrating pedagogical philosophy (macro), technological architecture (meso), and instructional implementation (micro). Specifically, at the macro level, it establishes the “AI-Enhanced Student-Led Tutorial Education” Teaching Model as a philosophical foundation to reshape the triadic interaction between teachers, students, and machines. The meso level centers on three technological cores—Knowledge Graph, AI, and Task Engines—which integrate to create personalized, immersive learning environments. Finally, at the micro level, the model implements a human-computer collaborative approach designed specifically to train clinical thinking skills. Furthermore, targeting the critical shortage of digitally-proficient faculty in Southwest China’s border regions, the study proposes a “Cloud-Empowered Lightweight Adoption” model designed to lower technical thresholds. The feasibility of this approach in bridging infrastructural gaps and empowering regional talent cultivation was validated through a practical case study at Kunming University. This research establishes a novel pedagogical paradigm that not only guides the reform of veterinary infectious disease education in Western China but also offers a scalable model for institutions worldwide facing similar resource constraints, thereby contributing to the global advancement of veterinary capacity building.

## 1 Introduction

To effectively meet the World Organisation for Animal Health (OIE)’s core requirements for member countries’ veterinary services, pedagogical design must strictly align with its competency-based standards. Therefore, the systematic construction of veterinary infectious disease courses serves not only as the cornerstone for cultivating practical professional skills but also as a strategic initiative to enhance national veterinary service capabilities and safeguard global public health security (1). However, the traditional teaching model for this course faces inherent bottlenecks. Abstract pathogenic mechanisms, complex epidemiological chains, and high-biosecurity-risk outbreak scenarios are difficult to present intuitively through conventional lecture-based methods, while structural constraints—such as large class sizes and limited practical resources—hinder effective clinical competency training (2). These challenges are exacerbated in the ecologically complex border regions of southwestern China, resulting in a significant gap between talent cultivation and regional demands for epidemic prevention and control.

Since the beginning of the 21st century, veterinary infectious disease education in China has gradually shifted from a “teacher-textbook-classroom” centered approach to an outcome-based education (OBE) model, characterized by modular integration, case-based learning (CBL), and problem-based learning (PBL) (3–5). This transition aligns with global trends, where international models—such as the organ-system integration in North America, the evidence-based clinical focus in Europe, and the biosecurity-centric training in Australasia—collectively emphasize “student-centered” learning and a “One Health” perspective (6–8). While these pedagogical philosophies provide critical benchmarks, their effective implementation is often hindered by the limitations of traditional instructional tools. Conventional methods struggle to fully simulate complex epidemiological scenarios or support personalized learning pathways, thereby constraining the depth of competency development.

The COVID-19 pandemic catalyzed a global digital transformation in education, rapidly accelerating the deep integration of big data, artificial intelligence, and virtual simulation into veterinary curricula (9, 10). Digitally and intelligently empowered teaching offers a novel pathway to bridge the aforementioned gaps—specifically, overcoming the limitations of traditional instruction and providing the advanced tools necessary for effective OBE implementation (11, 12). Therefore, to fully harness this potential and address the specific resource constraints in Southwest China, this study systematically explores the key roles, practical applications, and potential challenges of digital-intelligent technologies in veterinary infectious disease education. The ultimate aim is to construct a scalable teaching model that facilitates the paradigm shift from “knowledge transmission” to “competency development” tailored to regional needs.

## 2 The core philosophy and framework of digital-intelligent teaching

### 2.1 The paradigm shift from “teaching” to “learning”

The traditional teaching model, centered on the ‘teacher-textbook-classroom’ triad, relies on unidirectional knowledge transmission and standardized curricula supplemented by basic confirmatory experiments (13, 14). While efficient for building knowledge frameworks, this approach has significant limitations that are particularly pronounced in the resource-limited southwestern border regions. Firstly, teacher-student interaction is minimal, leading to delayed feedback and a stagnant classroom atmosphere. Secondly, this unidirectional instruction fosters passivity and prevents the vast majority of students from engaging in active and deep thinking. Furthermore, the overreliance on nationally unified teaching materials creates a serious disconnect with the unique disease ecology of the southwestern region, which is characterized by a “three-dimensional climate, multiple host species, and multiple epidemic focus (15–17). Abstract and complex pathogenic mechanisms and disease transmission chains cannot be visually presented, rendering the acquired knowledge inadequate for tackling locally prevalent cross-border animal epidemics. Additionally, constrained by biosafety requirements, costs, and spatiotemporal limitations, it is extremely difficult to replicate and practice within real-world scenarios of high-risk or large-scale animal disease outbreaks.

In contrast to these traditional limitations, the digital-intelligent teaching model constructs a new “intelligence-augmented” pedagogical paradigm centered on students, problems, and competencies (11). Within this framework, the teacher’s role transforms into a learning designer and guide. The teaching vehicle evolves from static textbooks into a dynamic and interactive digital foundation, comprising: (i) integrated knowledge graphs that visually map complex logical relationships; (ii) generative AI-driven smart case libraries that provide infinite, variable clinical scenarios; and (iii) immersive VR/AR training platforms that safely simulate high-risk outbreak environments. The teaching setting transcends the boundaries of the physical classroom, forming a blended learning space that integrates online and offline elements (18, 19). Its core strength lies in facilitating sustained, personalized interaction. AI learning companions provide immediate feedback on student’s thought processes, effectively stimulating engagement. Consequently, abstract content becomes tangible and practical training scalable, ensuring that theoretical knowledge is efficiently internalized into clinical reasoning skills.

The successful transition to this paradigm represents not a mere superimposition of technology, but the construction of a novel instructional architecture. This architecture is designed to shift students from passive reception to active inquiry, ultimately fostering critical thinking and clinical proficiency.

### 2.2 Construction of the core framework

To systematically address the core challenges in veterinary infectious disease education, digital-intelligent teaching establishes a framework driven by three core engines: the Knowledge Graph Engine, AI Engine, and Task Engine. Functioning complementarily, the Knowledge Graph structures the content, the AI Engine personalizes the interaction, and the Task Engine drives practical application. Together, they reshape student learning pathways, facilitating a leap from passive reception to active construction.

The Knowledge Graph Engine functions as the “knowledge brain,” transforming the vast, complex knowledge system of veterinary infectious diseases into a dynamic and visual network (20). By clarifying logical connections—such as how pathogen traits dictate control strategies—it shifts learning from fragmented memorization to a systematic perspective.

The AI Engine acts as the “intelligent heart” of the framework. It functions as an inexhaustible “teaching capability platform” through the deep integration of large language models with a rigorously validated, veterinary-specific knowledge base. This platform dynamically generates highly realistic smart cases that cover various diseases and complexity levels, all tailored to specific learning objectives and student proficiency (11). Furthermore, it simulates stakeholders (e.g., animal owners) for interactive consultations and serves as a feedback coach, providing real-time evaluation of diagnostic reasoning, thereby providing personalized scaffolding for learning (21).

The Task Engine is the “driving nerve” of the framework. Unlike conventional task-based learning, which often relies on static assignments and delayed feedback, this engine dynamically structures the learning process into adaptive, interlinked task sequences. Guided by the “learning-by-doing” philosophy, the engine leads students through a complete cycle—from receiving a case, to information collection, differential diagnosis, and plan development, culminating in summary and reflection. The AI Engine accompanies this entire process, ensuring each practical step receives immediate feedback and support, thereby transforming isolated knowledge points into solid, closed-loop clinical reasoning capability.

In summary, the three engines do not operate in isolation but form an organic whole: the Knowledge Graph Engine defines the learning territory and objectives, the AI Engine provides limitless personalized interactive scenarios and intelligent support, and the Task Engine drives students to complete the crucial leap from knowledge to competency within this intelligent “high-fidelity immersive environment.” Ultimately, this achieves precise and efficient cultivation of high-quality veterinary professionals.

## 3 Key technologies and practical applications

### 3.1 Knowledge graph

In the digital-intelligent teaching reform of veterinary infectious diseases, the knowledge graph serves as the “Graph Engine,” playing a pivotal role in the systematic reconstruction of the knowledge system. As a technological model, the knowledge graph is built upon an “entity-relationship-attribute” triplet structure, which formally defines and connects disparate knowledge points into a cohesive semantic network. To demonstrate the efficacy of this approach, we reference the implementation on a collaborative platform developed by Yangzhou University. This specific instance integrates 233 knowledge points and 149 relationships across four structured layers (resources, knowledge, problems, and competencies).^1^ Crucially, this structural methodology is platform-independent; it provides a replicable blueprint for any institution to construct proprietary course graphs tailored to their specific curriculum needs, provided compatible digital infrastructure is available.

### 3.2 AI large models and smart case systems

Within the digital-intelligent teaching practice of veterinary infectious diseases, AI large models and smart case systems, serving as the “AI Engine,” are profoundly reshaping the model for cultivating clinical competencies. This engine, grounded in a rigorously validated veterinary knowledge base, dynamically generates an endless supply of highly realistic smart cases, fundamentally overcoming the limitations of traditional physical case libraries in scale, diversity, and timeliness. However, given the potential for generative inaccuracies, continuous expert verification and a “human-in-the-loop” oversight mechanism remain essential to ensure clinical precision.

In practice, the AI-driven “Smart Case Library” enables on-demand generation of tailored scenarios across diverse species, disease stages, and farming models. Beyond static content, these cases feature dynamic evolution and multi-turn dialog capabilities, allowing students to engage in immersive consultations with AI-simulated stakeholders. The scenario evolves in real-time based on student decisions, precisely training information extraction skills. Exemplifying this, the “VIRT-Vet” system from Fudan University provides real-time evaluation of students’ decisions, pinpointing logical flaws while using guided questioning to develop their diagnostic reasoning.

### 3.3 Virtual simulation and VR/AR technologies

Within the digital-intelligent teaching system for veterinary infectious diseases, virtual simulation and VR/AR technologies effectively overcome the biosafety constraints and resource limitations of traditional practical training by creating highly immersive, zero-risk virtual operational environments (22, 23). Its application is primarily manifested in two core scenarios: In experimental teaching involving highly pathogenic pathogens, students can safely perform high-risk procedures in a virtual laboratory, mastering standard protocols without assuming any biosafety risks. In outbreak emergency management drills, the systems simulate the complete progression of a major animal epidemic, enabling students to adjust control strategies and visually observe the impact of different measures on disease spread, thereby cultivating their macro-level decision-making and crisis response capabilities. Ultimately, these virtual rehearsals translate into measurable learning outcomes, significantly enhancing students’ procedural accuracy and confidence before they engage in real-world clinical practice. Currently, Chinese universities have made significant progress in this field. Representative projects from Yangzhou University, China Jiliang University, and Huazhong Agricultural University have focused on virtual simulations for pathogenic detection, quarantine inspection, and viral isolation.^2^ As recognized National First-Class Undergraduate Courses, these projects fully demonstrate the significant value and potential for broader adoption of such technologies in teaching complex system simulations.

### 3.4 Intelligent teaching platforms and smart assessment

Intelligent teaching platforms facilitate digital transformation through an integrated architecture categorized into three functional layers: (i) access and resource delivery via learning portals; (ii) interactive support provided by AI learning companions; and (iii) precision evaluation through competency assessment systems. Together, these modules construct a complete “learning-practicing-assessment-application” pedagogical closed loop. Their innovativeness is primarily manifested in the smart assessment system: through formative evaluation functions, the AI system can comprehensively record and analyze each decision-making step students take during case discussions and virtual operations, providing precise diagnostics for their learning pathways (24, 25). Simultaneously, leveraging visualization tools like competency radar charts, it continuously tracks students’ developmental trajectories across multiple dimensions—including knowledge comprehension, clinical skills, and AI application. This granular analytics capability is fundamental to Competency-Based Education (CBE), as it shifts assessment from static scorekeeping to the dynamic verification of professional proficiency (26, 27).

Currently, the intelligent teaching platform ecosystem in Chinese higher education has developed into a multi-layered, complementary system, providing a solid foundation for digital-intelligent instruction in courses like veterinary infectious diseases. Foundational public platforms (e.g., Chaoxing, Zhihuishu) facilitate resource sharing, while specialized tools like “Rain Classroom” enhance real-time interaction (28, 29). Furthermore, the national “ilab-x” platform aggregates high-tier virtual experiments, enabling zero-risk drills for high-pathogenic outbreaks. Collectively, these platforms form a comprehensive support chain encompassing “basic applications – specialized tools – cutting-edge exploration – specialized breakthroughs,” offering diverse and implementable technological pathways for advancing teaching reform in universities across all regions and at various levels.

## 4 The holistic construction of the digital-intelligent teaching model

### 4.1 The macro-level educational philosophy of digital-intelligent teaching models

**Figure 1:**
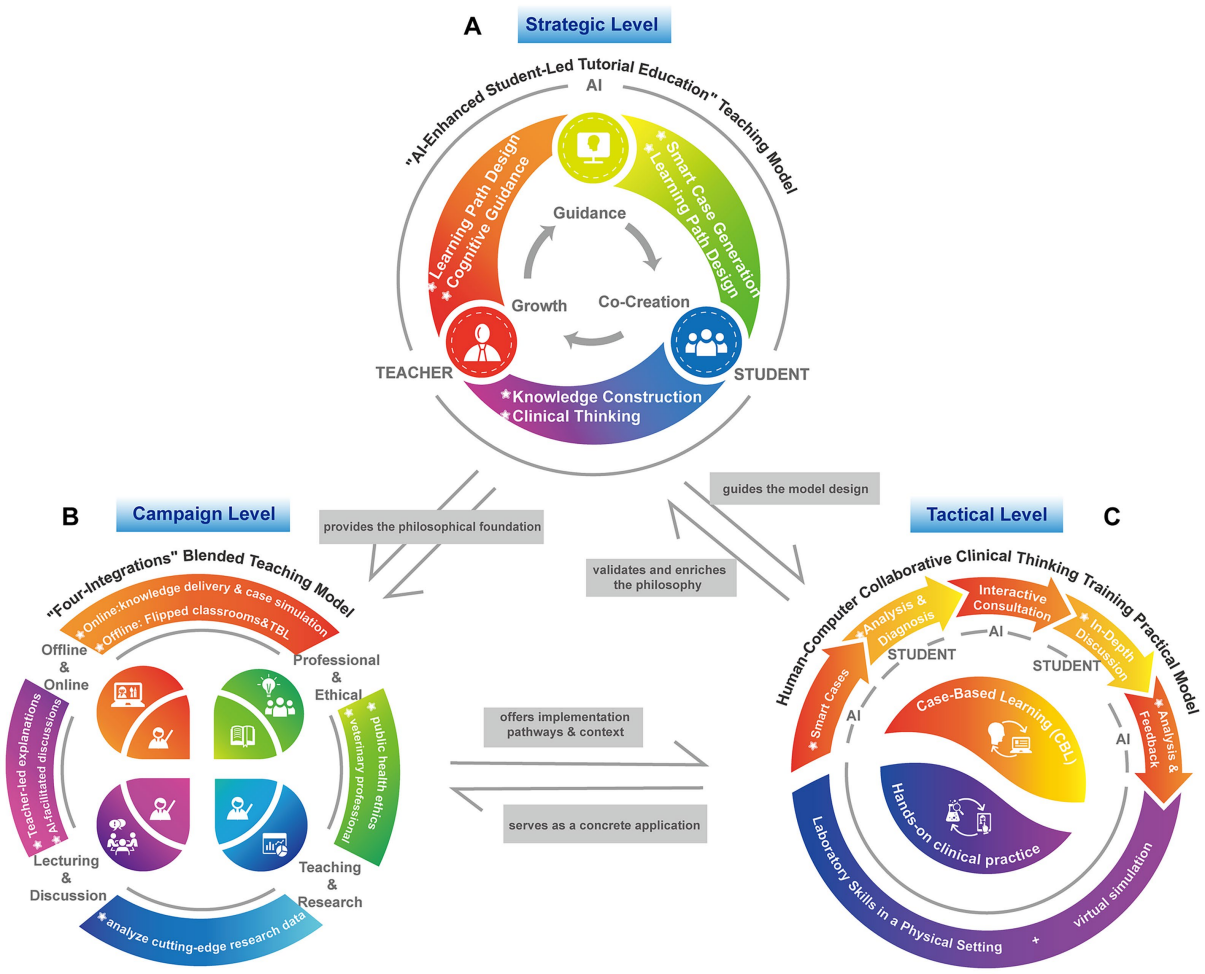
The architecture and internal logic of the digital-intelligent teaching model. **(A)** The “AI-Enhanced Student-Led Tutorial Education” teaching model, demonstrating the student-centered pedagogical relationship. **(B)** The “Four-Integrations” blended teaching model, illustrating the systematic integration of teaching dimensions. **(C)** The “Human-Computer Collaborative Clinical Thinking Training” practical model, showcasing the dynamic clinical decision-making process.

The “AI-Enhanced Student-Led Tutorial Education” model serves as the guiding philosophy for reshaping pedagogical relationships. As shown in Figure 1A, this model leverages AI not merely as a tool but as an intelligent partner, fundamentally shifting the dynamic from teacher-centric instruction to student-centric co-creation. Teachers evolve into pathway designers, while students actively construct knowledge through open-ended inquiry. This human-computer collaboration realizes a “guidance – co-creation – growth” closed loop. This model has not only strengthened students’ diagnostic decision-making and innovative thinking abilities but also prompted teachers to refresh their educational philosophies and iterate their mentoring approaches, thereby providing a transferable framework for cultivating veterinary talent in the new era.

### 4.2 The curriculum system development pathway for digital-intelligent teaching models

The “Four-Integrations” Blended Teaching Model reconstructs the teaching ecology of veterinary infectious diseases through multi-dimensional integration (Figure 1B). This model is structured on the foundational framework of Integrating Online and Offline learning. It utilizes AI platforms for knowledge delivery and case simulation, while employing offline flipped classrooms and Team-Based Learning (TBL) to achieve knowledge internalization and competency development. It is guided by the value orientation of integrating professional knowledge with ethics and responsibility, seamlessly incorporating elements of public health ethics and veterinary professional conduct into teaching cases to shape students’ sense of professional mission. The model extends capabilities through integrating research and teaching, using AI tools to analyze cutting-edge research data, guiding students to identify scientific questions and nurturing innovative thinking. Its implementation pathway is characterized by integrating lecturing and discussion, where concise teacher-led explanations of core concepts are synergistically combined with AI teaching assistant-facilitated discussions, achieving a unity of conceptual depth and breadth of thinking. Through the systematic combination of these four dimensions, the model forms a comprehensive educational system (30–33). Ultimately, this “Four-Integrations” framework transcends the sum of its parts; by synergizing knowledge impartation, competency cultivation, value shaping, and innovation literacy, it offers a holistic paradigm that uniquely bridges the gap between fragmented teaching techniques and comprehensive talent cultivation.

### 4.3 The digital-intelligent clinical training pathway in veterinary infectious diseases

Clinical diagnostic and decision-making ability serves as the cornerstone and core objective in cultivating talent in veterinary infectious diseases (34). The development of this high-order competency has traditionally relied on two pillars of clinical teaching: Case-Based Learning (CBL) (35) and hands-on clinical practice. Digital-intelligent technologies transform these two pillars by integrating AI-driven interactive cases with high-immersion virtual simulations (Figure 1C). This fusion establishes a complete “clinical thinking flywheel” that cycles from cognition to practice and back again via feedback, making the cultivation of diagnostic and decision-making abilities more systematic, efficient, and traceable.

### 4.4 The internal logic of the digital-intelligent teaching model

The digital-intelligent teaching model for veterinary infectious diseases proposed in this study is not a mere compilation of methods, but an organic system composed of a macro-level philosophy, a mid-level framework, and micro-level practices. This system positions the “AI-Enhanced Student-Led Tutorial Education” Teaching Model as its overarching design and meta-model, aiming to fundamentally reshape the power dynamics and subject relationships within teaching. The “Four Integrations” Blended Teaching Model provides a structured implementation pathway and ecological support for “Student-Led” learning by systematically integrating four key dimensions. Building upon this foundation, the “Human-Computer Collaborative Clinical Thinking Training” Model serves as a focused practical approach for cultivating the most core clinical diagnostic competency in veterinary education. These three components interlock seamlessly, collectively forming a complete closed loop that progresses from “educational philosophy” to “curriculum system” and finally to “competency training,” thereby providing a clear theoretical blueprint and practical guide for the systematic and profound advancement of teaching reform (Figure 1).

## 5 Constraints and breakthrough pathways for constructing a digital-intelligent teaching system for veterinary infectious diseases in universities of Southwest China

Among the constraints faced by universities in Southwest China, the shortage of digitally-proficient faculty remains the most critical bottleneck, followed by funding and technical limitations. To address these challenges, an integrated strategy is proposed for developing the “Three Engines.” This approach advocates an “Introduce–Digest–Adapt” model for knowledge graphs, incorporating externally developed resources while localizing content to reflect regional disease characteristics. For AI case systems, a “Cloud-Based Access, Localized Adaptation” strategy is recommended—subscribing to established platforms while building localized case datasets tailored to area-specific diseases. In virtual simulation, a “Lightweight Terminal + Cloud Rendering” setup lowers hardware barriers, allowing high-quality simulations to run via affordable devices. Crucially, this “borrow-and-adapt” pathway significantly lowers the technical threshold for faculty, allowing them to focus on pedagogical design rather than software development. Together, these pathways form an integrated, cost-efficient, and locally adaptive model for digital-intelligent teaching transformation under resource and personnel constraints.

## 6 Case study of digital-intelligent curriculum construction for veterinary infectious diseases in universities in Southwest China: Kunming university as an example

Guided by the digital-intelligent teaching model proposed in this paper, the Veterinary Medicine Program at Kunming University explored a feasible pathway for veterinary infectious diseases education in Southwest China through deep collaboration with Yangzhou University, establishing a teaching implementation plan tailored to regional realities. Leveraging the advanced curriculum system developed by Yangzhou University on the “Zhihuishu Platform,” and integrating the disease prevalence characteristics and student learning conditions of the southwest region, our program has developed a course model that “balances theory and practice while integrating online and offline” components.

**Figure 2:**
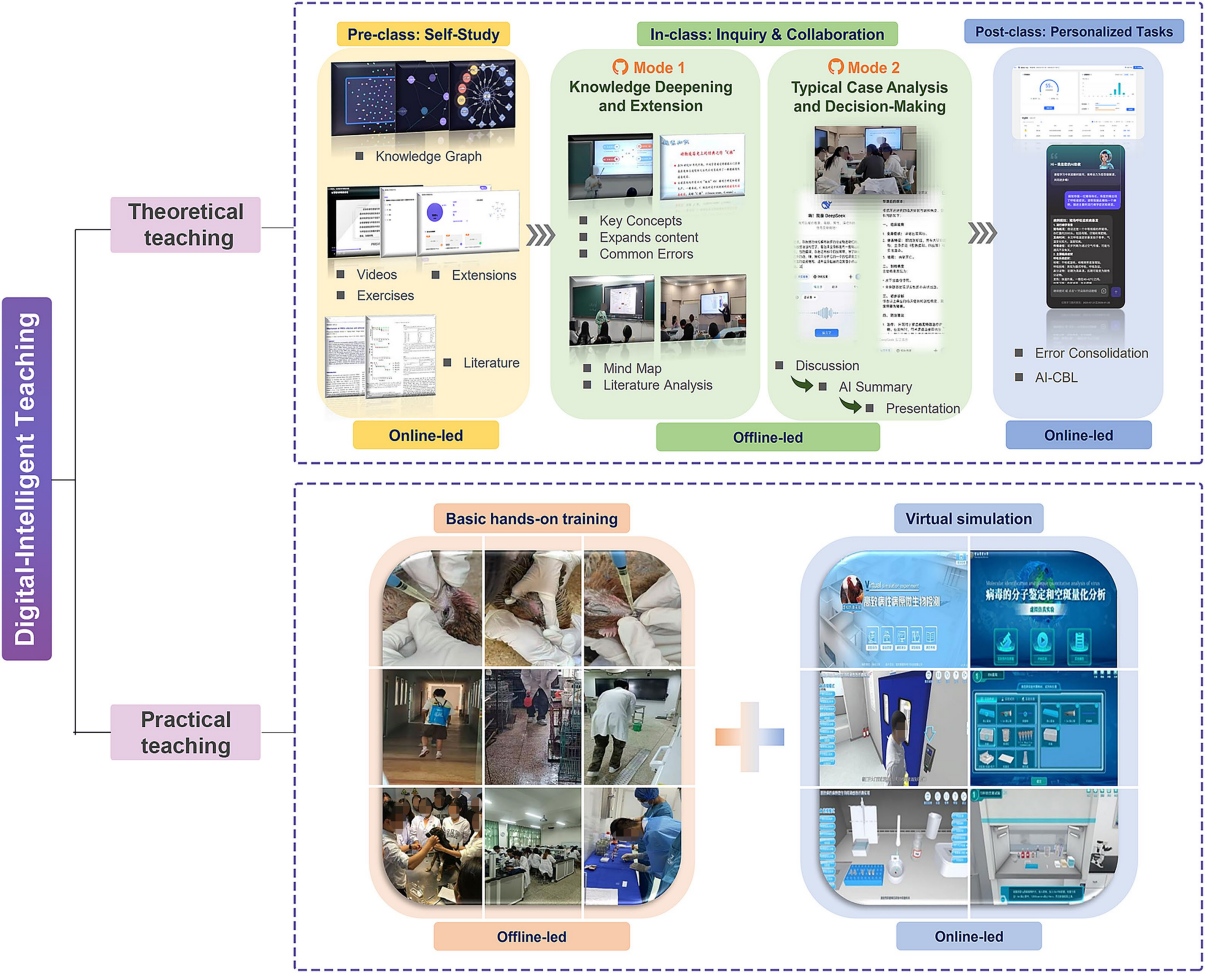
Digital-intelligent curriculum development model for veterinary infectious diseases at Kunming University. (Top) Flowchart of the theoretical instruction component, demonstrating the blended online-offline pedagogy that integrates modular knowledge systems with AI-assisted learning. (Bottom) Framework of the practical training component, illustrating the dual-track approach that combines hands-on skills training with virtual simulation exercises.

At the theoretical teaching level, the course adopts a blended online-offline approach. The online component centers on a “modular knowledge system – intelligent interactive system.” It systematically integrates knowledge points such as etiology and epidemiology through concise video lectures and knowledge graphs, dynamically embedding cases of regionally high-prevalence diseases. A “Large Language Model-based AI Teaching Assistant” is introduced to support intelligent Q&A and interactive diagnostic training, simulating consultation scenarios with an “AI Animal Owner” to guide students through complete clinical thinking exercises. Based on system-generated learning analytics, offline teaching is structured into two distinct instructional modes. The Knowledge Deepening and Extension Mode delivers focused lectures on key concepts, expands content based on regional cross-border disease control needs, and incorporates mind mapping demonstrations along with scientific literature data analysis to foster systematic thinking and evidence-based practice. Simultaneously, the Case Analysis and Decision-Making Mode is dedicated to organizing case study presentations and designing clinical scenario-based interactive exercises to strengthen diagnostic reasoning and address learning gaps (Figure 2).

At the practical teaching level, the course has established a dual-track model of “basic hands-on training + virtual simulation.” Offline practical sessions focus on training students in fundamental skills such as laboratory diagnostic methods for common diseases, animal vaccination techniques, and disinfection procedures. For high-risk, high-cost training content involving highly pathogenic pathogens and complex emergency outbreak response, virtual simulation experiments provide effective supplementation. Students can repeatedly practice the complete diagnosis and management procedures for major regional diseases like African Swine fever through the national Virtual Simulation Experiment Teaching Sharing Platform, all within a zero-biosafety-risk environment. This creates a new practical teaching paradigm where “basic skills are mastered offline, and advanced competencies are trained virtually” (Figure 2).

The course has also established a comprehensive, accompanying formative assessment system. Utilizing AI technology, it dynamically tracks student performance across all stages of theoretical learning and practical training, generating visual learning trajectories and competency profiles to achieve precise evaluation of students’ clinical thinking development and practical skills.

Application of this digital-intelligent curriculum system has yielded quantifiable educational gains. The average final examination score of the pilot cohort rose to 75.96, surpassing the 68.80 average of previous years. Moreover, a comprehensive competency analysis—covering political literacy, scientific literacy, professional competency, and global perspective—revealed a holistic improvement, with the overall course achievement value increasing from 0.77 to 0.81.

Ultimately, this case demonstrates that by adopting the “Cloud-Empowered Lightweight Adoption” model—characterized by a pathway of “Introduction, Adaptation, and Co-construction”—universities in Southwest China can construct a complete digital-intelligent teaching system despite resource constraints. This success establishes a replicable and scalable paradigm that holds significant reference value not only for regional universities across China but also for other resource-limited institutions worldwide seeking to implement high-quality veterinary education.

## 7 Discussion

To address the universal challenge of high-quality talent cultivation under resource constraints, this study moves beyond descriptive technological applications to construct a novel, clearly layered (macro-meso-micro) conceptual model for digital-intelligent teaching. Unlike previous approaches, the core novelty of this work lies not in the technology itself, but in the organic unification of student-centered constructivist philosophy with AI capabilities. It validates that intelligent technology serves as a key catalyst for reshaping the “teacher-student-machine” triad, thereby operationalizing the paradigm shift from “knowledge transmission” to “competency development.”

In the process of advancing digital-intelligent teaching for veterinary infectious diseases, it remains crucial to soberly recognize and address several key challenges. The most immediate concern involves AI-specific risks: model inaccuracies—often rooted in insufficient case datasets—demand meticulous model selection and training, while the risk of student overreliance necessitates robust teacher oversight and review mechanisms (27, 36–39). Beyond these technical concerns, institutional challenges persist, including the pressure on teachers to adapt to new roles and the need for long-term, evidence-based assessment frameworks to compare virtual versus physical learning outcomes, moving beyond mere subjective satisfaction surveys (40–42). Finally, at a foundational level, operational issues regarding data privacy, algorithmic fairness, and sustainable funding must be resolved to ensure the transition from technological application to deep educational reform (13, 43). Collectively, these challenges constitute critical barriers that must be overcome for digital-intelligent teaching to evolve from mere technological application toward a profound deepening of educational substance.

Looking ahead, digital-intelligent education in veterinary infectious diseases will undergo a profound transformation from “tool innovation” to “ecosystem reconstruction,” with the co-evolution of “AI+HI” (Artificial Intelligence + Human Intelligence) serving as the central thread running through this entire process (44). However, this evolution must be grounded in rigorous empirical validation. Future development will follow three trends: (i) Technological evolution will shift from efficiency-first intelligent tools toward “Responsible AI” embedded with algorithmic fairness and data privacy, while deeply integrating the “One Health” concept to cultivate a global public health perspective; (ii) Teaching models will advance to a new stage of human-machine bidirectional empowerment, where digital twin classrooms integration of AI’s computational precision with teachers’ humanistic care and creative thinking, establishing a future educational cornerstone rooted in rigorous empirical validation (45); (iii) Through co-construction and sharing of open platforms and standardized resource libraries, disciplinary and geographical boundaries will be broken down, leading to the formation of an inclusive, equitable, and continuously evolving global veterinary education ecosystem. This evolution represents a novel paradigm shift that transcends mere technological iteration to systematically reshape the educational ecosystem. Offering a scalable blueprint for global veterinary education, it lays a solid foundation for addressing future challenges of zoonotic diseases and safeguarding global biosecurity.

## Footnotes

1
https://wisdomh5.zhihuishu.com/course/index/1686315696688074752?courseId=1100000736&mapVersion=1

2
https://www.ilab-x.com/list3?sid=76
